# Brain functional connectivity patterns associated with symptoms of vestibular migraine

**DOI:** 10.3389/fnins.2023.1231273

**Published:** 2023-12-14

**Authors:** Xia Zhe, Hailian Zhang, Min Tang, Xiaoyan Lei, Xiaoling Zhang, Chenwang Jin

**Affiliations:** ^1^Department of Medical Imaging, The First Affiliated Hospital of Xi’an Jiaotong University, Xi’an, Shaanxi, China; ^2^Department of Radiology, The Fifth People's Hospital of Qinghai Province, Xining, Qinghai, China; ^3^Department of MRI, Shaanxi Provincial People’s Hospital, Xi’an, Shaanxi, China

**Keywords:** vestibular migraine, degree centrality, functional connectivity, default mode network, prefrontal cortex

## Abstract

**Background:**

Several functional magnetic resonance imaging (fMRI) investigations of patients with vestibular migraine (VM) have revealed abnormal functionality in different networks, indicating that VM is related to alterations in brain function. We sought to investigate the resting-state functional connectivity (FC) patterns during the interictal period in VM by combining data-driven voxel-wise degree centrality (DC) calculations and seed-based FC analyses, and thereby determine the associations between cerebral function and clinical symptoms.

**Methods:**

Thirty-eight patients with VM and 33 matched normal controls were recruited. DC was calculated and compared between the groups, and the FC of locations showing DC alterations was further tested using a seed-based technique. The participants’ clinical indicators were correlated with the DC and FC values of the brain areas.

**Results:**

In contrast to the control group, the VM group showed considerably lower DC values in the bilateral medial prefrontal cortex (mPFC) and significantly higher DC values in the right occipital lobe. In the seed-based FC analyses, patients with VM demonstrated fewer connections of the bilateral mPFC with the bilateral posterior cingulate cortex, right parahippocampus, right cerebellar posterior lobe, bilateral cuneus, and left precuneus. In addition, clinical data from patients, such as pain intensity, episode frequency, and the Dizziness Handicap Inventory score, were negatively related to these FC and DC impairments.

**Conclusion:**

Our findings showed changes in the default mode network and visual cortex in patients with VM, providing further insights into the complexity of the mechanisms underlying VM.

## Introduction

Vestibular migraine (VM) is a highly prevalent vestibular disorder characterized by recurrent head pain and attacks of vertigo along with vomiting, nausea, and hypersensitivity to auditory, visual, olfactory, and somatosensory stimuli (Lempert and von Brevern, 2019). VM represents a substantial healthcare burden (Formeister et al., 2018), and its diagnostic criteria have been recently published jointly by the International Headache Society and the Bárány Society [Headache Classification Committee of the International Headache Society (IHS), 2018]. VM is a new clinical entity, and the increasing attention from clinicians and scientists is expected to enhance research on this entity and thereby improve the quality of clinical care for patients with VM.

Advances in neuroimaging modalities have helped improve our understanding of the underlying mechanisms of VM. Some studies have proposed VM to be a central vestibular illness involving a complex neuronal network (Chen et al., 2021, 2023; Zhe et al., 2021, 2023; Li et al., 2022, 2023; Wang et al., 2023). Resting-state functional magnetic resonance imaging (fMRI) is a reliable and noninvasive method to investigate brain and central nervous system diseases. A few studies on VM have employed resting-state fMRI, with the findings indicating brain dysfunction in the regions involved in pain, vestibular processing, and multisensory integration. Recent resting-state studies have also proven that patients with VM display alterations in the amplitudes of low-frequency fluctuations (ALFF) and regional homogeneity (ReHo) in the brain areas involved in vestibular control of multisensory integration, including the temporal lobe and cerebellum (Liu et al., 2020; Li et al., 2023). Other studies have focused on functional connectivity (FC) in patients with VM, including the FC of the regions that participate in nociception with the vestibular and visual cortex regions (Chen et al., 2021; Wang et al., 2023). Using independent component analysis (ICA), VM has been connected to interrupted resting-state FC in multiple intrinsic neural networks, including the sensorimotor network, vestibular cortical network, visual network, and default mode network (DMN) (Li et al., 2022; Chen et al., 2023). However, the results of resting-state fMRI studies in patients with VM are intricate and inconclusive. Furthermore, the previous studies have not yielded defining specific neuroimaging biomarkers, presenting additional challenges in the treatment of patients with VM.

Voxel-wise assessments of degree centrality (DC) can characterize voxel centrality by evaluating the variety of connections between a given voxel and other voxels at the entire brain level, avoiding the subjectivity associated with seed-site selection and thereby ensuring high sensitivity, specificity, and test–retest reliability. High and low DC values in specific brain areas correspond to increased and decreased global connectivity, respectively (Zuo et al., 2012). In contrast to previous resting-state fMRI investigations that concentrated on regional functional measurements, i.e., ReHo (Li et al., 2023), ALFF (Liu et al., 2020; Li et al., 2023), ICA (Li et al., 2022; Chen et al., 2023), seed-based FC (Wang et al., 2023), and large-scale brain network analysis (Zhang et al., 2023), the index of voxel-wise DC can highlight the influence and significance of a given network at the voxel level and indicate the hub features of the functional brain network. This method has been extensively applied to survey DC abnormalities in relation to conditions such as trigeminal neuralgia (Liu et al., 2022; Ge et al., 2023), migraine (Zhang et al., 2017; Lee et al., 2019), and neuropathic pain (Zhang et al., 2022). However, the presence of DC abnormalities in patients with VM remains to be determined.

In the present investigation, we utilized a data-driven voxel-wise DC analysis and surveyed the intrinsic patterns of dysconnectivity across whole-brain functional networks in patients with VM. In order to identify the connections causing the differences in DC between patients with VM and healthy controls (HCs), seed-based FC analysis was performed on the basis of the voxel-wise DC findings. We hypothesized that patients with VM would show DC and FC changes in visual- and pain-processing-related regions, and that some of these changes may be associated with the severity of VM, which is reflected by the frequency and severity of VM headaches as well as the intensity of vertigo.

## Materials and methods

### Participants

The study population consisted of 38 right-handed patients with VM (32 without aura and six with aura) who presented to Shaanxi Provincial People’s Hospital, China between February 2017 and March 2023. The diagnosis of VM was performed by an expert neurologist with accordance with the criteria proposed in the ICHD-3 [Headache Classification Committee of the International Headache Society (IHS), 2018]. All individuals underwent magnetic resonance imaging (MRI) and standard neurological and neuro-otological examinations. All of the procedures were completed on the same day during no-symptom intervals. Vestibular-induced myogenic potential stimulation, video nystagmography, head-impulse tests, and caloric testing revealed no peripheral vestibular dysfunction. None of the patients were receiving any regular medication to prevent VM. To avoid any possible pharmacological interferences in the blood oxygen level-dependent (BOLD) signal changes, the patients with VM were asked to avoid taking any medication for at least three days prior to the scan.

The HC group consisted of 33 individuals enrolled from the community who were matched for handedness, age, and sex. We excluded participants showing mental, migraine, chronic pain, neurologic, or vestibular disorders as well as those with systemic disorders, stroke, secondary somatoform vertigo, drug abuse, or trauma. None of the patients showed white matter lesions or structural abnormalities on conventional MRI imaging. This study was approved by the Shaanxi Provincial People’s Hospital’s ethics committee. The participants provided written informed consent before study enrollment.

### Clinical assessment

All patients completed questionnaire assessments based on the Head Impact Test-6 (HIT-6), the Dizziness Handicap Inventory (DHI), the Visual Analog Scale (VAS), and the Migraine Disability Assessment Scale (MIDAS) (Sauro et al., 2010; Hawker et al., 2011; Balci et al., 2018). The patients’ sex, age, illness duration, and frequency of episodes were also recorded by the neurologist.

### Imaging data acquisition

A 3.0 T scanner (Philips Ingenia, Best, Netherlands) with a 16-channel head coil was used to obtain all images. A high-resolution 3D T1-weighted imaging scan covering the entire brain was obtained with the following imaging parameters: repetition time (TR)/echo time (TE), 1900/2.26 ms; flip angle, 9°; inversion time, 900 ms; slice thickness, 1.00 mm (with no interslice gap); matrix, 256 × 256; and field of view (FOV), 220 × 220 mm. Resting-state fMRI images were obtained by gradient echo-planar imaging with the following parameters: TR/TE, 2000/30 ms; slice thickness, 4 mm; slice gap, 0 mm; number of slices, 34; matrix, 128 × 128; FOV, 230 × 230 mm; flip angle, 90°; number of volumes, 200. Throughout the scan, the patients were asked to shut their eyes and stay alert and quiet in a resting posture. The patients were then questioned whether they were awake throughout the scan.

### Data preprocessing

Functional data were handled using the DPABI toolbox in MATLAB software (version 2014b) (Yan et al., 2016). Preprocessing involved the following steps: (1) To ensure stability of BOLD signal, the first ten time points were discarded. (2) Slice-timing correction was performed for interleaved acquisitions to correct the time delay between slices. (3) 3D head motion correction was performed, and participants with large head movements were excluded (data with maximum displacement in head rotation >2° or displacement larger than 2 mm in any direction were eliminated) (Yan et al., 2016). (4) Individual T1 data were registered to individual EPI templates and then segmented into three major tissue types: gray matter, white matter, and cerebrospinal fluid; the registration parameters obtained after cutting were used to register the individual EPI template to the standard Montreal Neurological Institute space, and the voxel size for resampling was 3 mm × 3 mm × 3 mm. (5) Twenty-four head motion parameters, cerebrospinal fluid signals, and white matter signals were removed from the equation reduce the influences of confounding factors. To minimize the impact of physiological noise originating from respiration and cardiac activity, a series of preprocessing steps were performed, including temporal band-pass filtering (0.01–0.08 Hz) and linear detrending.

### DC processing

The DPABI software was used to perform voxel-wise DC analysis. Initially, Pearson’s correlation coefficients between the time series of all pairs of gray matter (GM) voxels were computed to produce a whole-brain FC matrix for each participant. This computation was carried out under a GM mask created by thresholding (probability N > 0.2) the GM probability map in SPM12. Individual correlation matrices underwent a Fisher’s r-to-z transformation to derive the Z-score matrix to enhance normality. To generate a correlation matrix, Pearson’s correlations were computed for the time course from each voxel to every other voxel in the entire brain. The binarized matrix was created by thresholding each correlation at *r* > 0.25 (Zuo and Xing, 2014; Li et al., 2016). This threshold was the default value in the DC map calculation, and it was intended to prevent counting voxels with low temporal correlation due to signal noise. Finally, the DC maps were smoothed spatially with a 6-mm full-width at half-maximum (FWHM) Gaussian kernel.

### FC analysis

For FC analyses of spatial smoothing (FWHM = 6 mm) data, a seed-based approach was applied. The regions of interest (ROIs) were the brain areas demonstrating substantially changed DC in VM patients. These regions were usually identified as seeds. The time series of all voxels inside the seed area were averaged to obtain the time series for each ROI. Pearson’s correlation analyses were performed for each seed’s averaged time series and the remainder of the brain. A correlation map was prepared for each participant. Finally, Fisher’s z transformation was used to normalize the correlation map.

### Statistical analysis

#### Demographic and clinical data

The clinical data were examined by SPSS statistical software. A two-sample *t*-test was used to assess the age and education variables, and sex-related comparisons between patients with VM and HCs were performed using the chi-square test. The significance threshold was confirmed as *p* < 0.05.

#### DC and FC analysis

DC and FC maps were analyzed using a two-sample *t*-test in DPABI software to assess differences between the patients with VM and HCs, with age and sex as covariates. Multiple comparisons were amended using a Gaussian random approach (GRF-corrected, voxel *p* < 0.001, cluster *p* < 0.05). The surviving clusters were recorded.

#### Correlation analysis

The relationships of individual mean DC and FC values with clinical markers in patients with VM were evaluated by Pearson’s correlation analysis. Statistical significance was set at *p* < 0.05.

## Results

### Demographic and clinical features

The VM and HC groups did not significantly differ in terms of age, sex, or educational attainment (*p* > 0.05). The mean VAS, HIT-6, and MIDAS scores in the VM group were 5.07 ± 2.73, 57.71 ± 8.08, and 49.39 ± 40.25, respectively, indicating a moderate-to-severe migraine burden. The mean DHI score in the VM group was 44.32 ± 16.21, indicating moderate vertigo symptoms (Table 1).

**Table 1:** Demographic and clinical characteristics of the patients.

Characteristics	VM (*n* = 38) Mean ± SD	HCs (*n* = 33) Mean ± SD	*p* value
Sex (male/female)*	6/32	5/28	0.94
Age (years)	38.34 ± 11.70	38.15 ± 11.94	0.94
Education (years)	14.26 ± 3.39	14.87 ± 2.49	0.39
Disease duration (years)	7.76 ± 10.59		
VM episode frequency (number)	5.68 ± 3.51		
VAS score	5.07 ± 2.73		
MIDAS score	49.39 ± 40.25		
HIT-6 score	57.71 ± 8.08		
DHI score	44.32 ± 16.21		

*Chi-square test.

### Intergroup differences in DC

**Figure 1:**
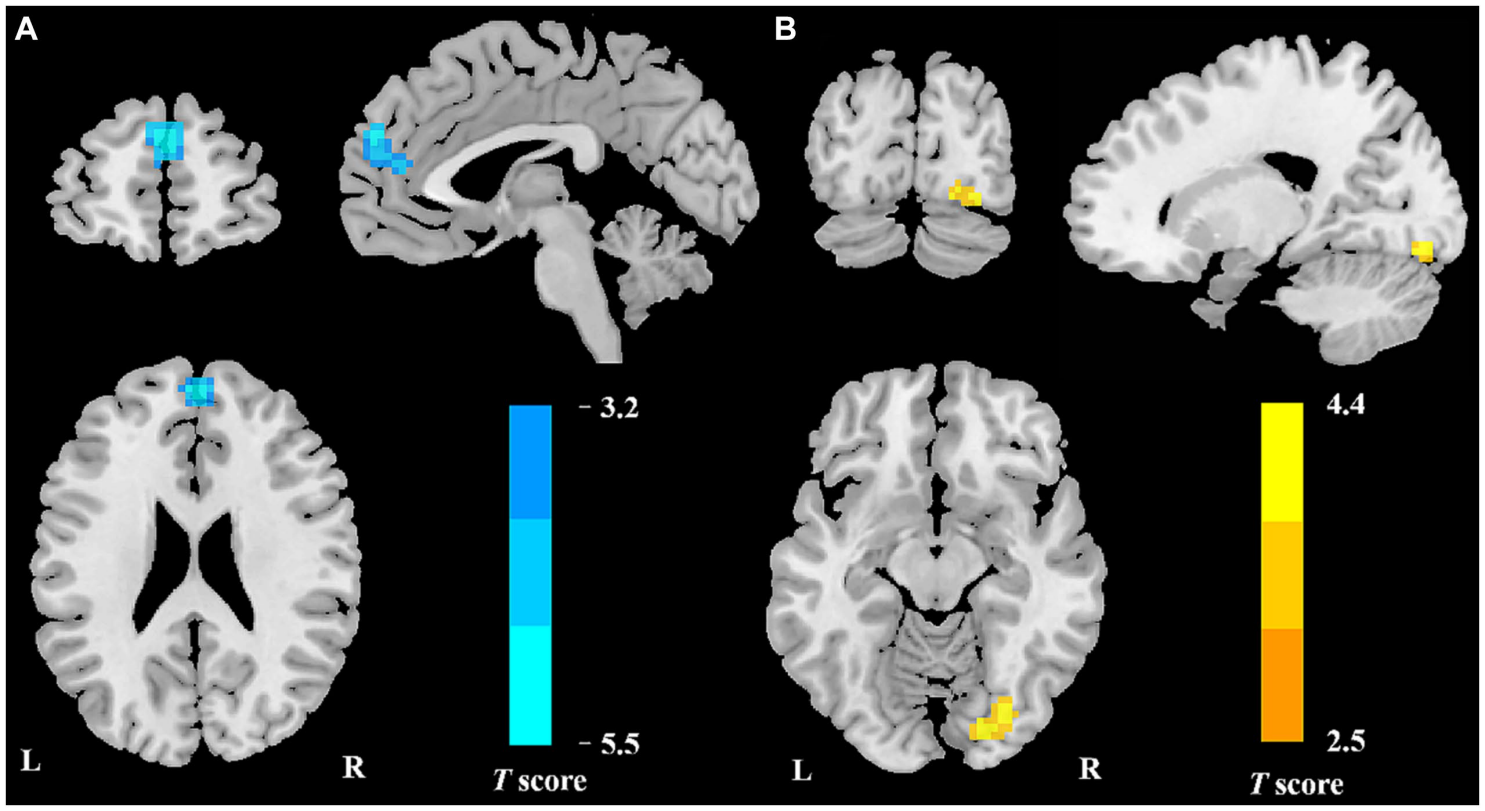
Regional DC reduction in patients with VM in contrast to HCs. In comparison with HCs, patients with VM showed significantly decreased DC values in the bilateral mPFC **(A)** and increased DC values in the right occipital lobe **(B)**.

Compared to HCs, patients with VM exhibited reduced DC values in the bilateral medial prefrontal cortex (mPFC) and increased DC values in the right occipital lobe (Table 2; Figure 1).

**Table 2:** DC differences between patients with VM and HCs.

Brain region		MNI			Voxels	*T*
		x	y	z		
mPFC	L	−3	−57	24	107	−5.520
mPFC	R	5	55	24	43	−3.233
Occipital lobe	R	18	−84	−12	75	4.443

### Intergroup differences in FC

**Figure 2:**
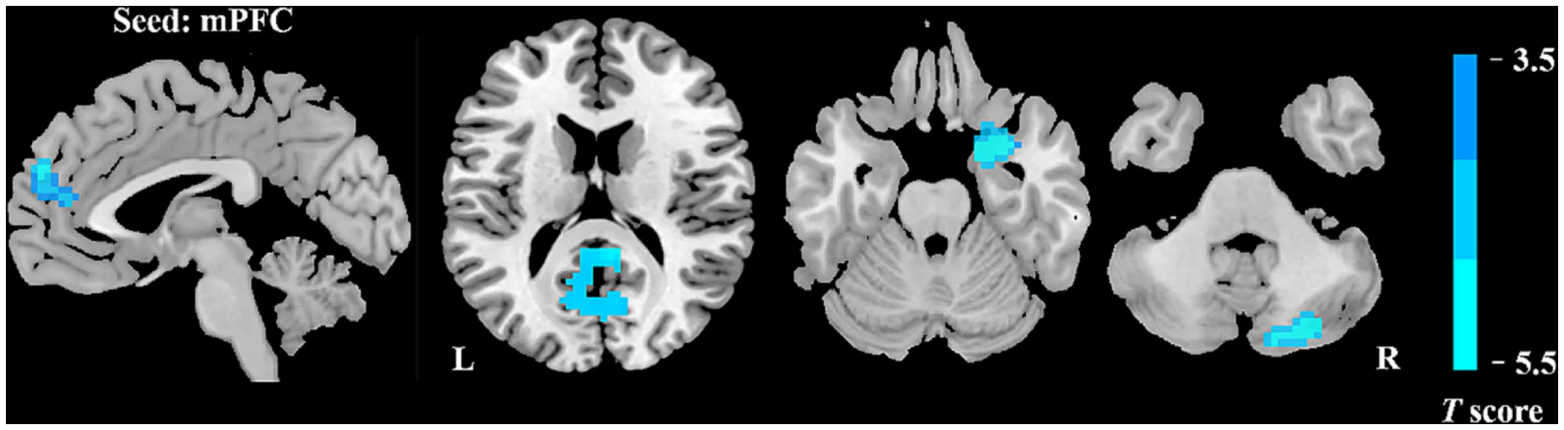
Abnormal FC of patients with VM in contrast to HCs. Relative to the control group, the VM group showed decreased FC of the bilateral mPFC with the bilateral posterior cingulate cortex (PCC), right parahippocampus, bilateral cuneus, left precuneus, and right cerebellar posterior lobe.

We further assessed seed-based FC in relation to two ROIs (the bilateral mPFC and the right occipital lobe). In comparison with HCs, patients with VM showed remarkably lower resting-state FC between the mPFC and multiple brain regions, including the bilateral posterior cingulate cortex (PCC), right parahippocampus, bilateral cuneus, left precuneus, and right cerebellar posterior lobe (Figure 2; Table 3). The two groups showed no significant differences in the right occipital lobe and other brain locations.

**Table 3:** FC differences between patients with VM and HCs.

Seed points	Brain region		MNI			Voxels	*T*
			X	y	z		
L/R mPFC	Posterior cingulate	L	−6	−48	3	159	−5.370
	Posterior cingulate	R	5	−45	15	29	−5.370
	Parahippocampus	R	30	6	−24	99	−5.402
	Precuneus	L	−3	−75	27	127	−4.434
	Cuneus	L	−45	−81	−3	95	−4.055
	Cuneus	R	12	−70	23	26	−4.434
	Cerebellar posterior lobe	R	24	−81	−39	94	−4.688

### Correlation analysis

**Figure 3:**
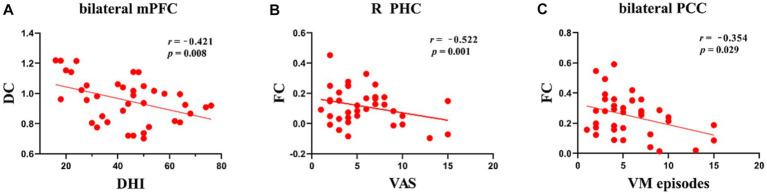
Scatter plots of correlations between brain function and clinical variables in patients with VM. **(A)** Negative correlations were observed between DC values in the bilateral mPFC and DHI scores, *p* < 0.05. **(B)** Negative correlations were observed between FC of the mPFC and parahippocampus and VAS scores, *p* < 0.05. **(C)** Negative correlations were observed between FC of the mPFC and bilateral PCC and VM episodes, *p* < 0.05.

Patients with VM showed a negative connection between DC values in the mPFC and the DHI score (*r* = −0.421, *p* = 0.008; Figure 3). In patients with VM, the FC between the bilateral mPFC and the right parahippocampus (*r* = −0.522, *p* = 0.001; Figure 3) demonstrated a negative correlation with the VAS score, while the FC between the bilateral mPFC and the bilateral PCC (*r* = −0.354, *p* = 0.029; Figure 3) correlated negatively with VM frequency.

## Discussion

In comparison with HCs, patients with VM showed higher DC values for the bilateral mPFC and lower DC values for the right occipital lobe. Furthermore, seed-to-whole-brain voxel analyses displayed wide reductions in the FC between the mPFC and cortical regions, which included those involved in the visual cortex, limbic areas, and DMN. These functional abnormalities are associated with visual processing and failure in pain regulation and management. The results of correlation analyses suggested that a lower FC was substantially associated with the influence of frequent VM episodes on everyday living. The present findings yielded fresh insights into the neural mechanisms underlying the development of VM.

The mPFC and occipital lobe were altered in the DC evaluations in this study. The mPFC is usually thought to adjust weakening of pain perception by top-down cognitive control (Etkin et al., 2011). Our study proved that patients with VM exhibited lower DC values in the bilateral mPFC. Abnormal regional activity in the mPFC has been documented in patients with VM, which is consistent with our findings (Li et al., 2023). Our previous study on patients with VM also showed structural alterations in the mPFC in comparison with HCs (Zhe et al., 2020). In this study, the mPFC showed a strong negative correlation with the DHI score, suggesting that patients with VM who experienced more headaches each month showed lower activity in the mPFC. On the basis of these findings, the lower level of regional functional neural activity in the mPFC of VM patients can be considered to have contributed to the less effective control of pain perception. The occipital lobe is related to the visual cortex and plays a significant role in the pathophysiology of VM, which is the original region of cortical spreading depression (CSD) (Charles and Baca, 2013). In the present investigation, the occipital lobe of patients with VM and HCs showed differences in DC values. Consistent with these findings, previous investigations employing functional imaging of the brain have revealed hypermetabolism in the occipital lobe in patients with VM during the vertigo-free time (Shin et al., 2014). Thus, the higher DC values in the occipital lobe may represent vision-related perceptual disorders (for example, photophobia, afterimages, and visual snow). These study results may indirectly support the idea that CSD is the pathophysiology underpinning VM.

Furthermore, in the seed-based FC analysis, patients with VM showed lower FC between the bilateral mPFC and various brain areas, mainly the bilateral PCC, right parahippocampus, left precuneus, bilateral cuneus, and right cerebellar posterior lobe. The precuneus, mPFC, and PCC belong to the DMN (Raichle, 2015), which is thought to be a resting-state network essential for cognitive and pain processes (Buckner et al., 2008; Soheili-Nezhad et al., 2019). Multiple lines of evidence have demonstrated that the DMN nodes are chief hubs that intrinsically propagate brain activity and are usually more susceptible to pathological conditions (Deshpande et al., 2011; Garg et al., 2011). Our findings for these two regions indicated high levels of interference in the intrinsic activity propagation in the DMN in patients with VM. The PCC is known to be the core part of the DMN (Raichle, 2015). Some investigations have suggested that the DMN, including the PCC, may be engaged in the process of pain suppression and its efficacy (Nahman-Averbuch et al., 2014; Youssef et al., 2016). The PCC showed an evidently negative correlation with the frequency in this investigation. These results have shown that the PCC may accumulate damage due to repeated attacks of VM. This theory may explain the decreased FC between the mPFC and PCC. Therefore, the more severe the symptoms, the greater the disruption in FC. The precuneus has been linked to pain sensitivity (Qin et al., 2020; Rolls et al., 2020a,b). Emerson et al. (2014) demonstrated a substantial correlation between pain sensitivity and pain-induced reactions in the precuneus in HCs. Moreover, another study showed that pain sensitivity varies depending on the headache frequency (Pan et al., 2020). Thus, alterations in FC between the precuneus and mPFC may lead to enhanced pain sensitivity, which could induce hypersensitivity to external sensory stimuli. In brief, our results further indicated the presence of DMN dysfunction in patients with VM. The cuneus, which is located in the occipital lobe, is a part of the visual association cortex, is involved in the processing of visual spatial information, and has been connected to the processing of pain-related stimuli (Fortin et al., 2002; Waberski et al., 2008; Roy et al., 2012). The VM group showed weakened FC between the cuneus and mPFC, which is involved in the integration of visual information. Dysfunction of this area may cause abnormal integration of visual information.

In comparison with HCs, patients with VM also have a lower FC between the mPFC and the parahippocampus. Previous studies also demonstrated that the mPFC derives information from the thalamus and limbic system, and that it modulates pain response directly as well as indirectly by altering and modulating emotion and cognition (Ong et al., 2019; Thompson and Neugebauer, 2019). We speculate that patients with VM show reduced function of modulating the pain that may be in a sensitive state. The parahippocampus lies within the medial temporal lobe, which plays a role in memory processing (Insausti et al., 1998), and has also been linked to emotion (Smith et al., 2004) and visual and auditory inputs (Engelien et al., 2006; Arnott et al., 2008). Concurrent FC alterations in the parahippocampus and mPFC may be responsible for the common symptoms of VM, such as unpleasant memories, photophobia, and phonophobia. In our study, a lower FC between the two clusters was negatively associated with VAS scores, suggesting that painful experiences can trigger unpleasant memories, and that the development of these memories will further worsen the pain experience, which may be one of the causes of pain recurrences in VM patients.

Our study also showed reduced FC between the mPFC and right cerebellar posterior lobe in patients with VM relative to the findings in HCs. The cerebellum, which serves as a part of the pain matrix, can transfer pain information uphill via the fiber link with the cerebral cortex control by altering the brainstem nociceptive downward inhibitory route (Naegel et al., 2014). The compensation in vestibular rehabilitation that can effectively ameliorate vestibular symptoms is mainly mediated by the cerebellum (Liu et al., 2020). In terms of its functions in emotional and cognitive processing, the cerebellum shows physical linkages with different sections of the frontal cortex and limbic regions (Schmahmann and Caplan, 2006). Our study suggested that the disruption in the FC between the cerebellum posterior lobe and mPFC in individuals with VM may impede the transmission of pain information between the cerebellum and the cortex. These findings highlight the need for further research to determine the involvement of the cerebellum in the pathophysiology of VM.

## Limitations

Although our study showed that patients with VM exhibited deficits in FC between DMN regions and other cortical areas, the present study had some limitations. First, the sample size of this study was quite limited. Second, the patients were examined only in the interictal phase, indicating the need to evaluate functional abnormalities in the ictal phase. Third, the majority of patients with VM were female, and the potential influence of sex-related variations on the DC and FC alterations could not be ruled out.

## Conclusion

In summary, we used DC analysis in conjunction with seed-based FC data obtained from resting-state MRI to probe whole-brain FC in patients with VM. Our findings indicated that patients with VM displayed lower DC values in the mPFC and greater DC values in the right occipital lobe. Some of the regions with changes in DC demonstrated aberrant FC with other brain areas in the DMN and limbic areas linked with visual processing and dysfunction in the control and modulation of pain. The patients’ clinical information, such as episode frequency, DHI score, and pain intensity, were adversely connected to these FC and DC impairments, suggesting that a lower FC is strongly related to the influence of recurrent migraine episodes on daily life. Overall, our data indicate that patients with VM have distinct whole-brain FC abnormalities, which may support the occurrence of recurrent episodes in VM and present an additional treatment target.

## Data availability statement

The original contributions presented in the study are included in the article/supplementary material, further inquiries can be directed to the corresponding author.

## Ethics statement

The studies involving humans were approved by the Shaanxi Provincial People’s Hospital’s ethics committee. The studies were conducted in accordance with the local legislation and institutional requirements. The participants provided their written informed consent to participate in this study. Written informed consent was obtained from the individual(s) for the publication of any potentially identifiable images or data included in this article.

## Author contributions

XZ wrote the primary draft, devised the basic experiment, and completed the requisite statistical analysis. XL and XLZ assessed crucial clinical indicators. HZ collected the radical data. MT provided some technical support. CJ was responsible for study supervision and coordination. All authors contributed to the article, reviewed, and approved the submitted version.
